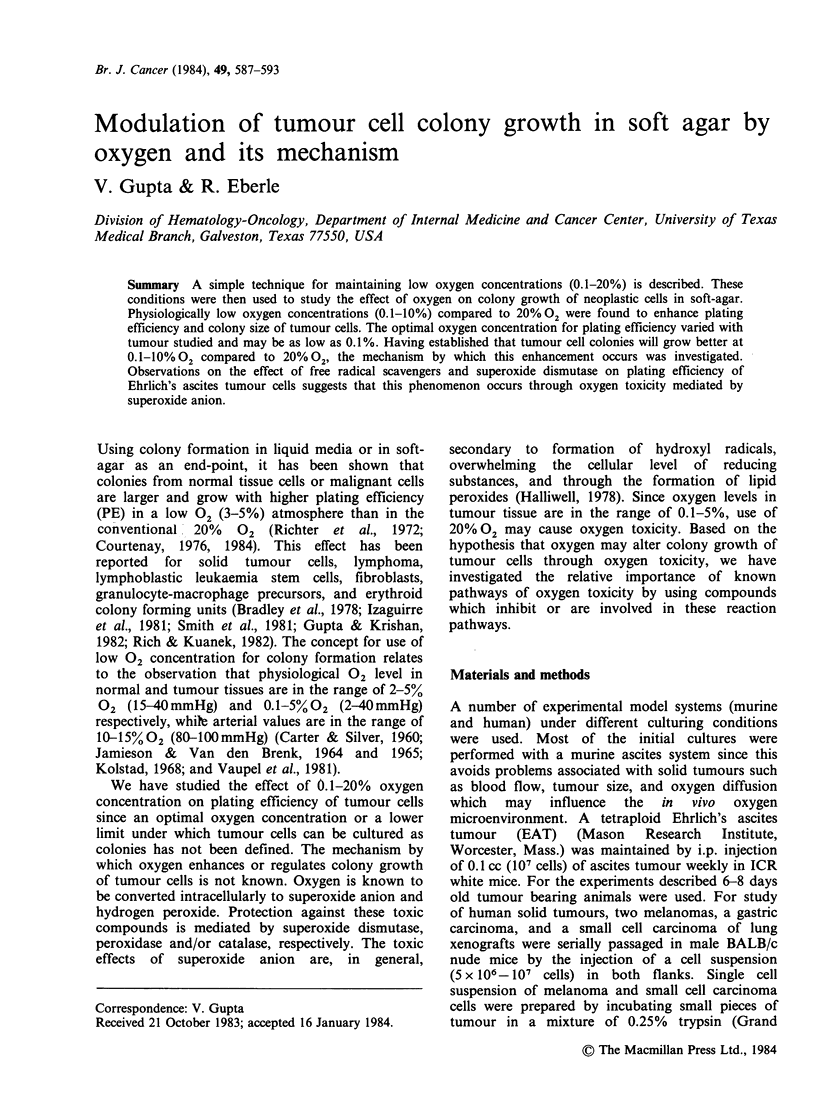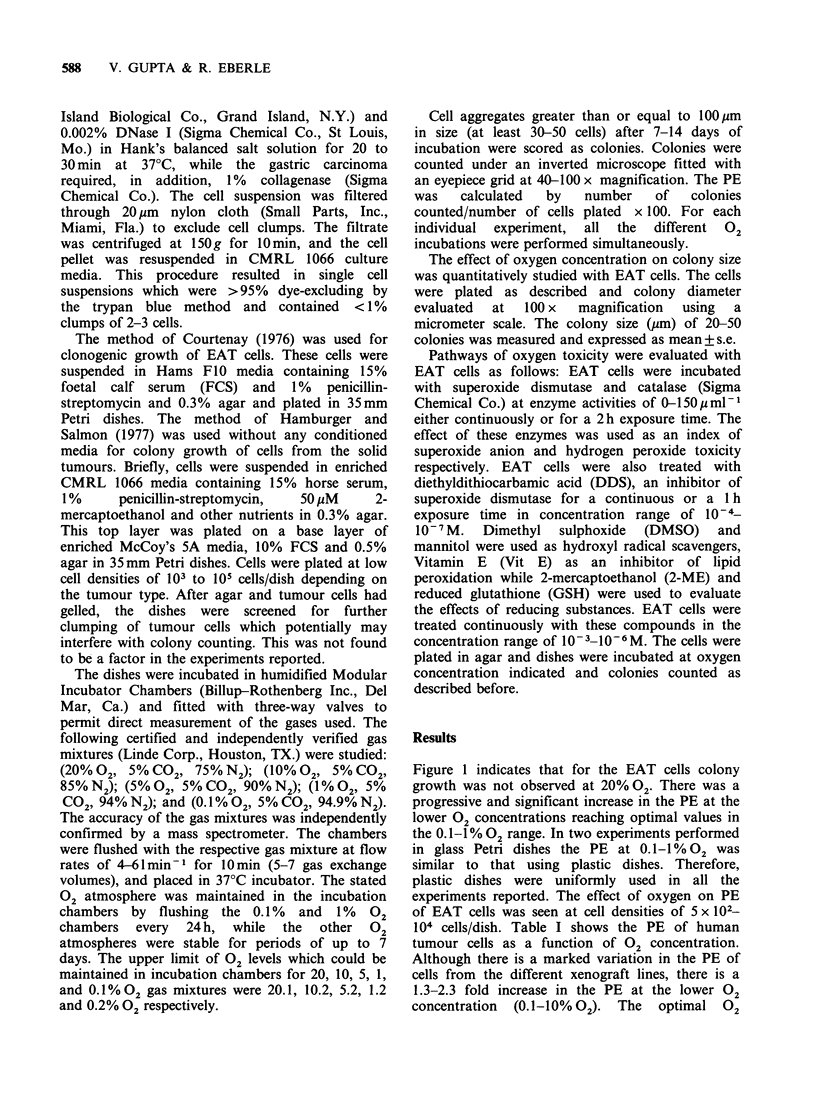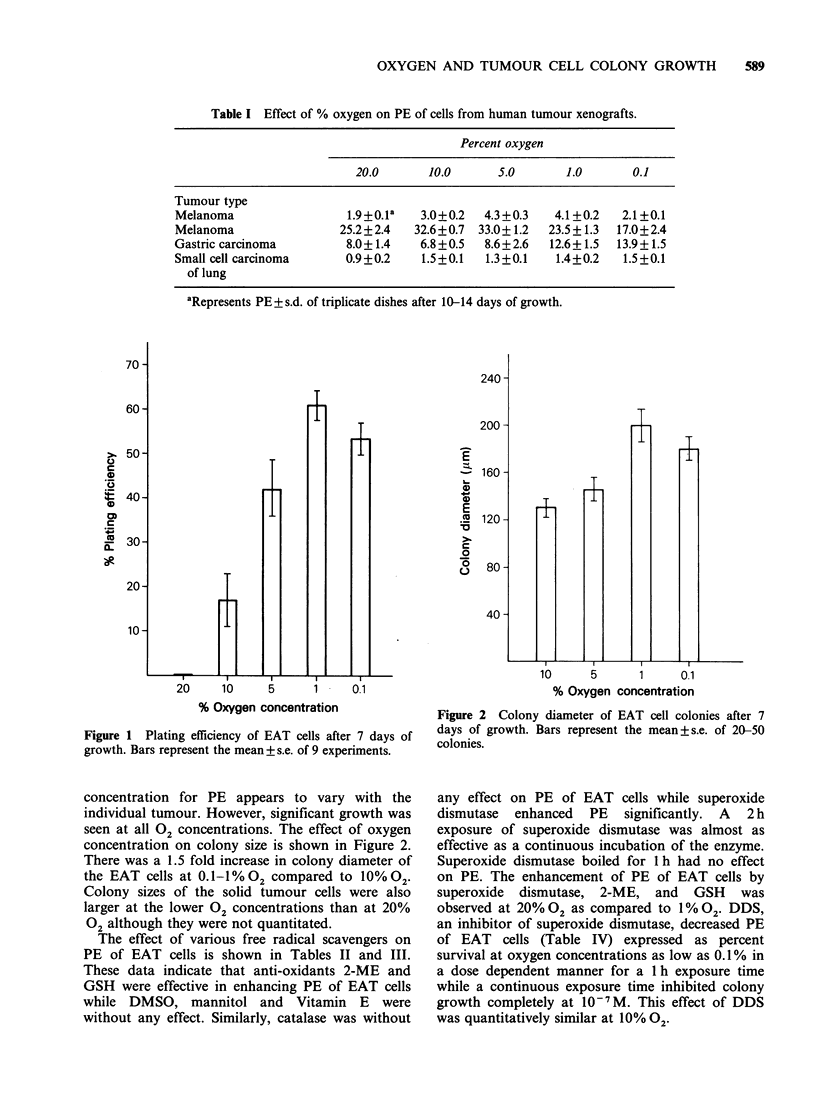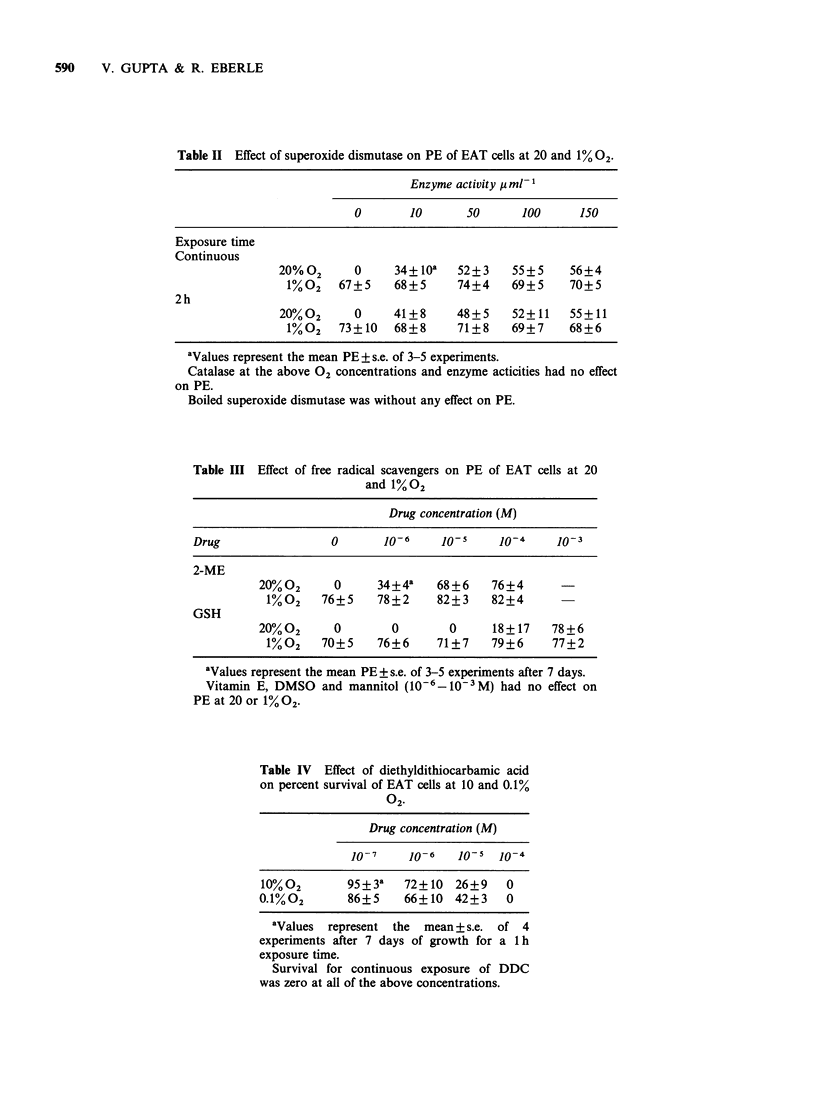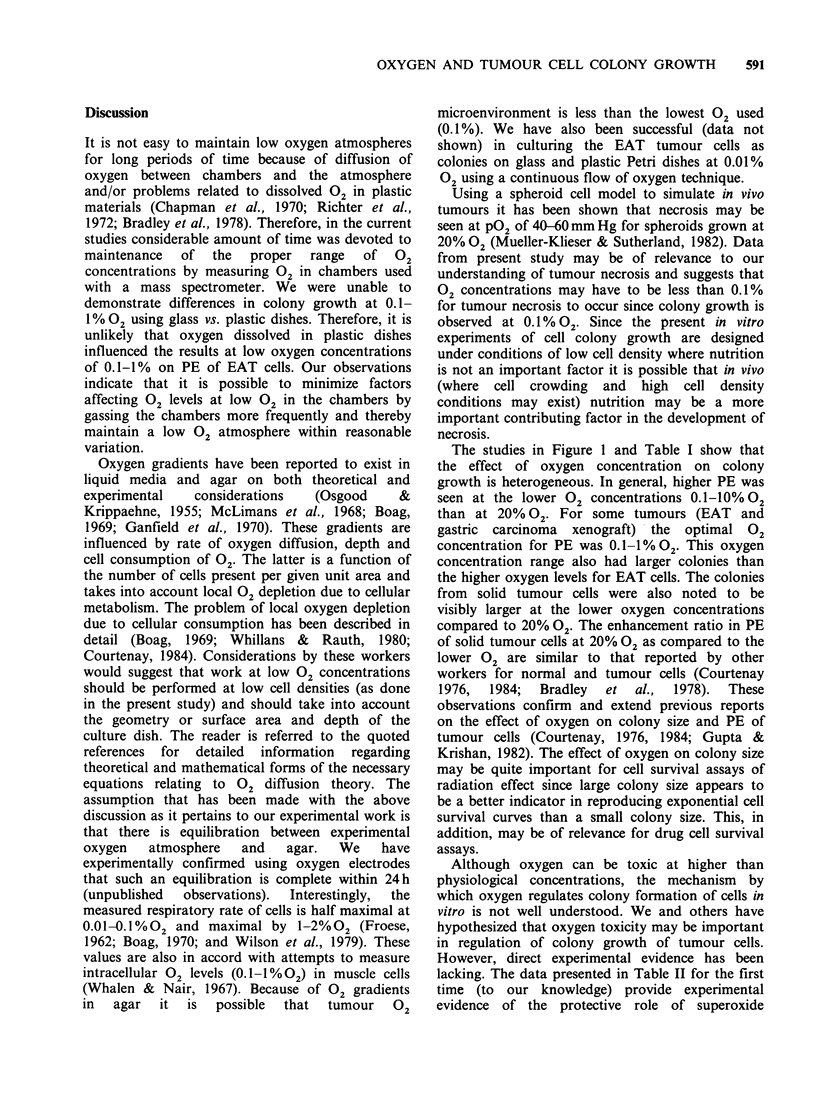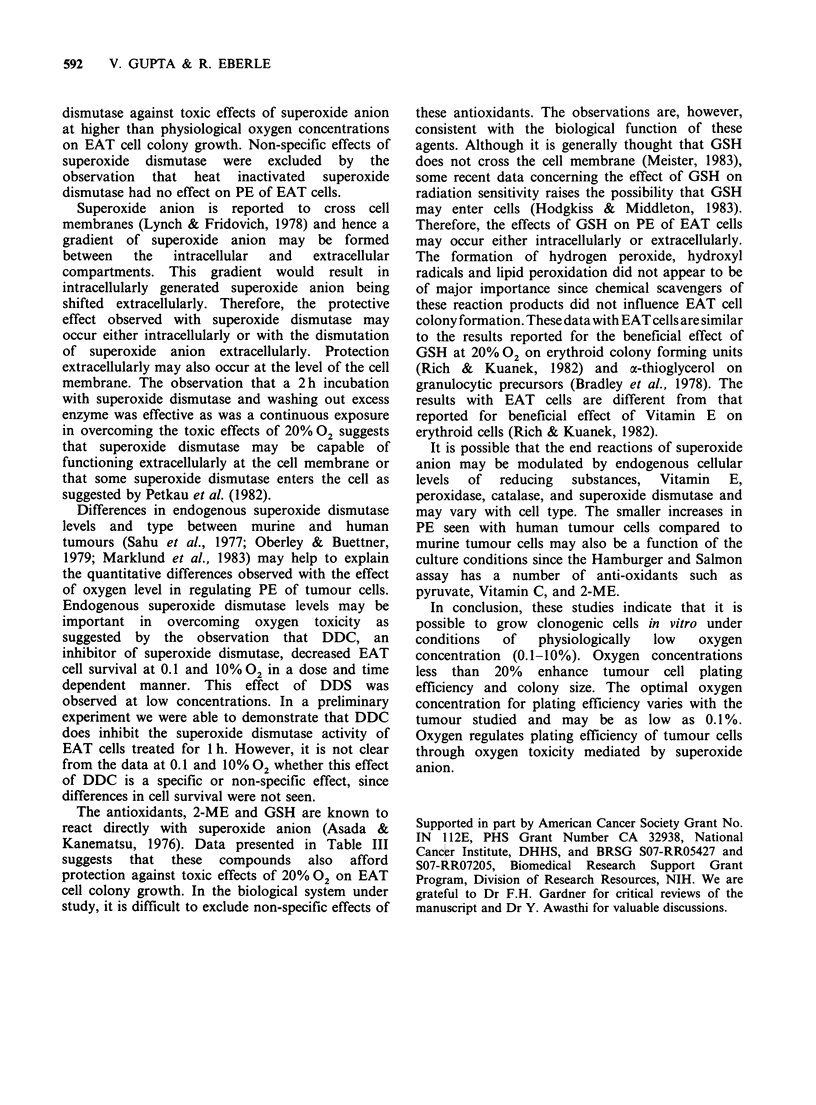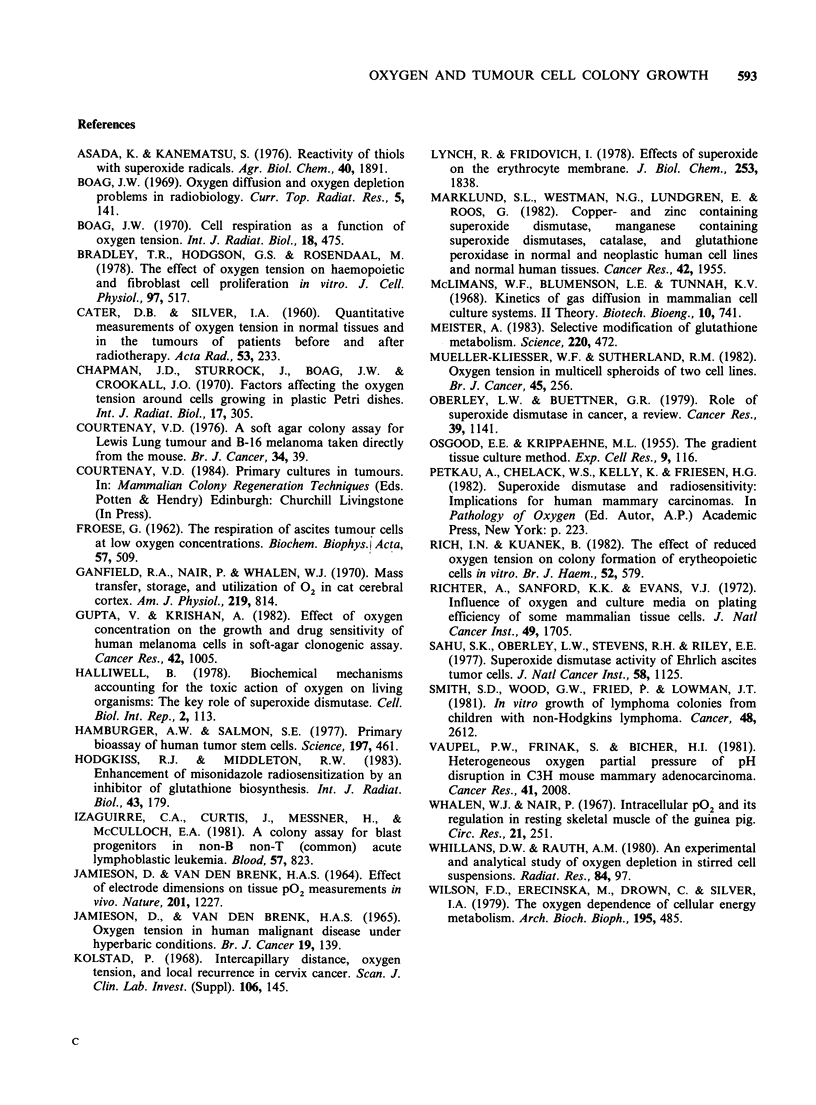# Modulation of tumour cell colony growth in soft agar by oxygen and its mechanism.

**DOI:** 10.1038/bjc.1984.93

**Published:** 1984-05

**Authors:** V. Gupta, R. Eberle

## Abstract

A simple technique for maintaining low oxygen concentrations (0.1-20%) is described. These conditions were then used to study the effect of oxygen on colony growth of neoplastic cells in soft-agar. Physiologically low oxygen concentrations (0.1-10%) compared to 20% O2 were found to enhance plating efficiency and colony size of tumour cells. The optimal oxygen concentration for plating efficiency varied with tumour studied and may be as low as 0.1%. Having established that tumour cell colonies will grow better at 0.1-10% O2 compared to 20% O2, the mechanism by which this enhancement occurs was investigated. Observations on the effect of free radical scavengers and superoxide dismutase on plating efficiency of Ehrlich's ascites tumour cells suggests that this phenomenon occurs through oxygen toxicity mediated by superoxide anion.


*Br. J. Cancer* (1984), **49**, 587–593

### Modulation of tumour cell colony growth in soft agar by oxygen and its mechanism

V. Gupta & R. Eberle

*Division of Hematology-Oncology, Department of Internal Medicine and Cancer Center, University of Texas Medical Branch, Galveston, Texas 77550, USA*

**Summary** A simple technique for maintaining low oxygen concentrations (0.1–20%) is described. These conditions were then used to study the effect of oxygen on colony growth of neoplastic cells in soft-agar. Physiologically low oxygen concentrations (0.1–10%) compared to 20% $O_2$ were found to enhance plating efficiency and colony size of tumour cells. The optimal oxygen concentration for plating efficiency varied with tumour studied and may be as low as 0.1%. Having established that tumour cell colonies will grow better at 0.1–10% $O_2$ compared to 20% $O_2$, the mechanism by which this enhancement occurs was investigated. Observations on the effect of free radical scavengers and superoxide dismutase on plating efficiency of Ehrlich's ascites tumour cells suggests that this phenomenon occurs through oxygen toxicity mediated by superoxide anion.

Using colony formation in liquid media or in soft-agar as an end-point, it has been shown that colonies from normal tissue cells or malignant cells are larger and grow with higher plating efficiency (PE) in a low $O_2$ (3–5%) atmosphere than in the conventional 20% $O_2$ (Richter *et al.*, 1972; Courtenay, 1976, 1984). This effect has been reported for solid tumour cells, lymphoma, lymphoblastic leukaemia stem cells, fibroblasts, granulocyte-macrophage precursors, and erythroid colony forming units (Bradley *et al.*, 1978; Izaguirre *et al.*, 1981; Smith *et al.*, 1981; Gupta & Krishan, 1982; Rich & Kuanek, 1982). The concept for use of low $O_2$ concentration for colony formation relates to the observation that physiological $O_2$ level in normal and tumour tissues are in the range of 2–5% $O_2$ (15–40 mmHg) and 0.1–5% $O_2$ (2–40 mmHg) respectively, while arterial values are in the range of 10–15% $O_2$ (80–100 mmHg) (Carter & Silver, 1960; Jamieson & Van den Brenk, 1964 and 1965; Kolstad, 1968; and Vaupel *et al.*, 1981).

We have studied the effect of 0.1–20% oxygen concentration on plating efficiency of tumour cells since an optimal oxygen concentration or a lower limit under which tumour cells can be cultured as colonies has not been defined. The mechanism by which oxygen enhances or regulates colony growth of tumour cells is not known. Oxygen is known to be converted intracellularly to superoxide anion and hydrogen peroxide. Protection against these toxic compounds is mediated by superoxide dismutase, peroxidase and/or catalase, respectively. The toxic effects of superoxide anion are, in general, secondary to formation of hydroxyl radicals, overwhelming the cellular level of reducing substances, and through the formation of lipid peroxides (Halliwell, 1978). Since oxygen levels in tumour tissue are in the range of 0.1–5%, use of 20% $O_2$ may cause oxygen toxicity. Based on the hypothesis that oxygen may alter colony growth of tumour cells through oxygen toxicity, we have investigated the relative importance of known pathways of oxygen toxicity by using compounds which inhibit or are involved in these reaction pathways.

### Materials and methods

A number of experimental model systems (murine and human) under different culturing conditions were used. Most of the initial cultures were performed with a murine ascites system since this avoids problems associated with solid tumours such as blood flow, tumour size, and oxygen diffusion which may influence the *in vivo* oxygen microenvironment. A tetraploid Ehrlich's ascites tumour (EAT) (Mason Research Institute, Worcester, Mass.) was maintained by i.p. injection of 0.1 cc ($10^7$ cells) of ascites tumour weekly in ICR white mice. For the experiments described 6–8 days old tumour bearing animals were used. For study of human solid tumours, two melanomas, a gastric carcinoma, and a small cell carcinoma of lung xenografts were serially passaged in male BALB/c nude mice by the injection of a cell suspension ($5 \times 10^6 - 10^7$ cells) in both flanks. Single cell suspension of melanoma and small cell carcinoma cells were prepared by incubating small pieces of tumour in a mixture of 0.25% trypsin (Grand

Correspondence: V. Gupta
Received 21 October 1983; accepted 16 January 1984.

© The Macmillan Press Ltd., 1984

Island Biological Co., Grand Island, N.Y.) and 0.002% DNase I (Sigma Chemical Co., St Louis, Mo.) in Hank's balanced salt solution for 20 to 30 min at 37°C, while the gastric carcinoma required, in addition, 1% collagenase (Sigma Chemical Co.). The cell suspension was filtered through 20 $\mu$m nylon cloth (Small Parts, Inc., Miami, Fla.) to exclude cell clumps. The filtrate was centrifuged at 150 $g$ for 10 min, and the cell pellet was resuspended in CMRL 1066 culture media. This procedure resulted in single cell suspensions which were >95% dye-excluding by the trypan blue method and contained <1% clumps of 2–3 cells.

The method of Courtenay (1976) was used for clonogenic growth of EAT cells. These cells were suspended in Hams F10 media containing 15% foetal calf serum (FCS) and 1% penicillin-streptomycin and 0.3% agar and plated in 35 mm Petri dishes. The method of Hamburger and Salmon (1977) was used without any conditioned media for colony growth of cells from the solid tumours. Briefly, cells were suspended in enriched CMRL 1066 media containing 15% horse serum, 1% penicillin-streptomycin, 50 $\mu$M 2-mercaptoethanol and other nutrients in 0.3% agar. This top layer was plated on a base layer of enriched McCoy's 5A media, 10% FCS and 0.5% agar in 35 mm Petri dishes. Cells were plated at low cell densities of $10^3$ to $10^5$ cells/dish depending on the tumour type. After agar and tumour cells had gelled, the dishes were screened for further clumping of tumour cells which potentially may interfere with colony counting. This was not found to be a factor in the experiments reported.

The dishes were incubated in humidified Modular Incubator Chambers (Billup–Rothenberg Inc., Del Mar, Ca.) and fitted with three-way valves to permit direct measurement of the gases used. The following certified and independently verified gas mixtures (Linde Corp., Houston, TX.) were studied: (20% $O_2$, 5% $CO_2$, 75% $N_2$); (10% $O_2$, 5% $CO_2$, 85% $N_2$); (5% $O_2$, 5% $CO_2$, 90% $N_2$); (1% $O_2$, 5% $CO_2$, 94% $N_2$); and (0.1% $O_2$, 5% $CO_2$, 94.9% $N_2$). The accuracy of the gas mixtures was independently confirmed by a mass spectrometer. The chambers were flushed with the respective gas mixture at flow rates of 4–6 l min$^{-1}$ for 10 min (5–7 gas exchange volumes), and placed in 37°C incubator. The stated $O_2$ atmosphere was maintained in the incubation chambers by flushing the 0.1% and 1% $O_2$ chambers every 24 h, while the other $O_2$ atmospheres were stable for periods of up to 7 days. The upper limit of $O_2$ levels which could be maintained in incubation chambers for 20, 10, 5, 1, and 0.1% $O_2$ gas mixtures were 20.1, 10.2, 5.2, 1.2 and 0.2% $O_2$ respectively.

Cell aggregates greater than or equal to 100 $\mu$m in size (at least 30–50 cells) after 7–14 days of incubation were scored as colonies. Colonies were counted under an inverted microscope fitted with an eyepiece grid at 40–100 × magnification. The PE was calculated by number of colonies counted/number of cells plated × 100. For each individual experiment, all the different $O_2$ incubations were performed simultaneously.

The effect of oxygen concentration on colony size was quantitatively studied with EAT cells. The cells were plated as described and colony diameter evaluated at 100 × magnification using a micrometer scale. The colony size ($\mu$m) of 20–50 colonies was measured and expressed as mean ± s.e.

Pathways of oxygen toxicity were evaluated with EAT cells as follows: EAT cells were incubated with superoxide dismutase and catalase (Sigma Chemical Co.) at enzyme activities of 0–150 $\mu$ ml$^{-1}$ either continuously or for a 2 h exposure time. The effect of these enzymes was used as an index of superoxide anion and hydrogen peroxide toxicity respectively. EAT cells were also treated with diethyldithiocarbamic acid (DDS), an inhibitor of superoxide dismutase for a continuous or a 1 h exposure time in concentration range of $10^{-4}$–$10^{-7}$ M. Dimethyl sulphoxide (DMSO) and mannitol were used as hydroxyl radical scavengers, Vitamin E (Vit E) as an inhibitor of lipid peroxidation while 2-mercaptoethanol (2-ME) and reduced glutathione (GSH) were used to evaluate the effects of reducing substances. EAT cells were treated continuously with these compounds in the concentration range of $10^{-3}$–$10^{-6}$ M. The cells were plated in agar and dishes were incubated at oxygen concentration indicated and colonies counted as described before.

### Results

Figure 1 indicates that for the EAT cells colony growth was not observed at 20% $O_2$. There was a progressive and significant increase in the PE at the lower $O_2$ concentrations reaching optimal values in the 0.1–1% $O_2$ range. In two experiments performed in glass Petri dishes the PE at 0.1–1% $O_2$ was similar to that using plastic dishes. Therefore, plastic dishes were uniformly used in all the experiments reported. The effect of oxygen on PE of EAT cells was seen at cell densities of $5 \times 10^2$–$10^4$ cells/dish. Table I shows the PE of human tumour cells as a function of $O_2$ concentration. Although there is a marked variation in the PE of cells from the different xenograft lines, there is a 1.3–2.3 fold increase in the PE at the lower $O_2$ concentration (0.1–10% $O_2$). The optimal $O_2$

**Table I** Effect of % oxygen on PE of cells from human tumour xenografts.

| | *Percent oxygen* | | | | |
|---|---|---|---|---|---|
| | *20.0* | *10.0* | *5.0* | *1.0* | *0.1* |
| Tumour type | | | | | |
| Melanoma | $1.9 \pm 0.1$[a] | $3.0 \pm 0.2$ | $4.3 \pm 0.3$ | $4.1 \pm 0.2$ | $2.1 \pm 0.1$ |
| Melanoma | $25.2 \pm 2.4$ | $32.6 \pm 0.7$ | $33.0 \pm 1.2$ | $23.5 \pm 1.3$ | $17.0 \pm 2.4$ |
| Gastric carcinoma | $8.0 \pm 1.4$ | $6.8 \pm 0.5$ | $8.6 \pm 2.6$ | $12.6 \pm 1.5$ | $13.9 \pm 1.5$ |
| Small cell carcinoma of lung | $0.9 \pm 0.2$ | $1.5 \pm 0.1$ | $1.3 \pm 0.1$ | $1.4 \pm 0.2$ | $1.5 \pm 0.1$ |

[a]Represents PE ± s.d. of triplicate dishes after 10–14 days of growth.

**Figure 1** Plating efficiency of EAT cells after 7 days of growth. Bars represent the mean ± s.e. of 9 experiments.

**Figure 2** Colony diameter of EAT cell colonies after 7 days of growth. Bars represent the mean ± s.e. of 20–50 colonies.

concentration for PE appears to vary with the individual tumour. However, significant growth was seen at all $O_2$ concentrations. The effect of oxygen concentration on colony size is shown in Figure 2. There was a 1.5 fold increase in colony diameter of the EAT cells at 0.1–1% $O_2$ compared to 10% $O_2$. Colony sizes of the solid tumour cells were also larger at the lower $O_2$ concentrations than at 20% $O_2$ although they were not quantitated.

The effect of various free radical scavengers on PE of EAT cells is shown in Tables II and III. These data indicate that anti-oxidants 2-ME and GSH were effective in enhancing PE of EAT cells while DMSO, mannitol and Vitamin E were without any effect. Similarly, catalase was without any effect on PE of EAT cells while superoxide dismutase enhanced PE significantly. A 2 h exposure of superoxide dismutase was almost as effective as a continuous incubation of the enzyme. Superoxide dismutase boiled for 1 h had no effect on PE. The enhancement of PE of EAT cells by superoxide dismutase, 2-ME, and GSH was observed at 20% $O_2$ as compared to 1% $O_2$. DDS, an inhibitor of superoxide dismutase, decreased PE of EAT cells (Table IV) expressed as percent survival at oxygen concentrations as low as 0.1% in a dose dependent manner for a 1 h exposure time while a continuous exposure time inhibited colony growth completely at $10^{-7}$ M. This effect of DDS was quantitatively similar at 10% $O_2$.

**Table II** Effect of superoxide dismutase on PE of EAT cells at 20 and 1% $O_2$.

| | | *Enzyme activity μml$^{-1}$* | | | | |
|---|---|---|---|---|---|---|
| | | *0* | *10* | *50* | *100* | *150* |
| Exposure time Continuous | | | | | | |
| | 20% $O_2$ | 0 | 34±10[a] | 52±3 | 55±5 | 56±4 |
| | 1% $O_2$ | 67±5 | 68±5 | 74±4 | 69±5 | 70±5 |
| 2 h | | | | | | |
| | 20% $O_2$ | 0 | 41±8 | 48±5 | 52±11 | 55±11 |
| | 1% $O_2$ | 73±10 | 68±8 | 71±8 | 69±7 | 68±6 |

[a]Values represent the mean PE±s.e. of 3–5 experiments.

Catalase at the above $O_2$ concentrations and enzyme acticities had no effect on PE.

Boiled superoxide dismutase was without any effect on PE.

**Table III** Effect of free radical scavengers on PE of EAT cells at 20 and 1% $O_2$

| | | *Drug concentration (M)* | | | | |
|---|---|---|---|---|---|---|
| *Drug* | | *0* | *$10^{-6}$* | *$10^{-5}$* | *$10^{-4}$* | *$10^{-3}$* |
| 2-ME | | | | | | |
| | 20% $O_2$ | 0 | 34±4[a] | 68±6 | 76±4 | — |
| | 1% $O_2$ | 76±5 | 78±2 | 82±3 | 82±4 | — |
| GSH | | | | | | |
| | 20% $O_2$ | 0 | 0 | 0 | 18±17 | 78±6 |
| | 1% $O_2$ | 70±5 | 76±6 | 71±7 | 79±6 | 77±2 |

[a]Values represent the mean PE±s.e. of 3–5 experiments after 7 days.

Vitamin E, DMSO and mannitol ($10^{-6}-10^{-3}$ M) had no effect on PE at 20 or 1% $O_2$.

**Table IV** Effect of diethyldithiocarbamic acid on percent survival of EAT cells at 10 and 0.1% $O_2$.

| | *Drug concentration (M)* | | | |
|---|---|---|---|---|
| | *$10^{-7}$* | *$10^{-6}$* | *$10^{-5}$* | *$10^{-4}$* |
| 10% $O_2$ | 95±3[a] | 72±10 | 26±9 | 0 |
| 0.1% $O_2$ | 86±5 | 66±10 | 42±3 | 0 |

[a]Values represent the mean±s.e. of 4 experiments after 7 days of growth for a 1 h exposure time.

Survival for continuous exposure of DDC was zero at all of the above concentrations.

### Discussion

It is not easy to maintain low oxygen atmospheres for long periods of time because of diffusion of oxygen between chambers and the atmosphere and/or problems related to dissolved $O_2$ in plastic materials (Chapman *et al.*, 1970; Richter *et al.*, 1972; Bradley *et al.*, 1978). Therefore, in the current studies considerable amount of time was devoted to maintenance of the proper range of $O_2$ concentrations by measuring $O_2$ in chambers used with a mass spectrometer. We were unable to demonstrate differences in colony growth at 0.1–1% $O_2$ using glass *vs.* plastic dishes. Therefore, it is unlikely that oxygen dissolved in plastic dishes influenced the results at low oxygen concentrations of 0.1–1% on PE of EAT cells. Our observations indicate that it is possible to minimize factors affecting $O_2$ levels at low $O_2$ in the chambers by gassing the chambers more frequently and thereby maintain a low $O_2$ atmosphere within reasonable variation.

Oxygen gradients have been reported to exist in liquid media and agar on both theoretical and experimental considerations (Osgood & Krippaehne, 1955; McLimans *et al.*, 1968; Boag, 1969; Ganfield *et al.*, 1970). These gradients are influenced by rate of oxygen diffusion, depth and cell consumption of $O_2$. The latter is a function of the number of cells present per given unit area and takes into account local $O_2$ depletion due to cellular metabolism. The problem of local oxygen depletion due to cellular consumption has been described in detail (Boag, 1969; Whillans & Rauth, 1980; Courtenay, 1984). Considerations by these workers would suggest that work at low $O_2$ concentrations should be performed at low cell densities (as done in the present study) and should take into account the geometry or surface area and depth of the culture dish. The reader is referred to the quoted references for detailed information regarding theoretical and mathematical forms of the necessary equations relating to $O_2$ diffusion theory. The assumption that has been made with the above discussion as it pertains to our experimental work is that there is equilibration between experimental oxygen atmosphere and agar. We have experimentally confirmed using oxygen electrodes that such an equilibration is complete within 24 h (unpublished observations). Interestingly, the measured respiratory rate of cells is half maximal at 0.01–0.1% $O_2$ and maximal by 1–2% $O_2$ (Froese, 1962; Boag, 1970; and Wilson *et al.*, 1979). These values are also in accord with attempts to measure intracellular $O_2$ levels (0.1–1% $O_2$) in muscle cells (Whalen & Nair, 1967). Because of $O_2$ gradients in agar it is possible that tumour $O_2$ microenvironment is less than the lowest $O_2$ used (0.1%). We have also been successful (data not shown) in culturing the EAT tumour cells as colonies on glass and plastic Petri dishes at 0.01% $O_2$ using a continuous flow of oxygen technique.

Using a spheroid cell model to simulate *in vivo* tumours it has been shown that necrosis may be seen at $pO_2$ of 40–60 mm Hg for spheroids grown at 20% $O_2$ (Mueller-Klieser & Sutherland, 1982). Data from present study may be of relevance to our understanding of tumour necrosis and suggests that $O_2$ concentrations may have to be less than 0.1% for tumour necrosis to occur since colony growth is observed at 0.1% $O_2$. Since the present *in vitro* experiments of cell colony growth are designed under conditions of low cell density where nutrition is not an important factor it is possible that *in vivo* (where cell crowding and high cell density conditions may exist) nutrition may be a more important contributing factor in the development of necrosis.

The studies in Figure 1 and Table I show that the effect of oxygen concentration on colony growth is heterogeneous. In general, higher PE was seen at the lower $O_2$ concentrations 0.1–10% $O_2$ than at 20% $O_2$. For some tumours (EAT and gastric carcinoma xenograft) the optimal $O_2$ concentration for PE was 0.1–1% $O_2$. This oxygen concentration range also had larger colonies than the higher oxygen levels for EAT cells. The colonies from solid tumour cells were also noted to be visibly larger at the lower oxygen concentrations compared to 20% $O_2$. The enhancement ratio in PE of solid tumour cells at 20% $O_2$ as compared to the lower $O_2$ are similar to that reported by other workers for normal and tumour cells (Courtenay 1976, 1984; Bradley *et al.*, 1978). These observations confirm and extend previous reports on the effect of oxygen on colony size and PE of tumour cells (Courtenay, 1976, 1984; Gupta & Krishan, 1982). The effect of oxygen on colony size may be quite important for cell survival assays of radiation effect since large colony size appears to be a better indicator in reproducing exponential cell survival curves than a small colony size. This, in addition, may be of relevance for drug cell survival assays.

Although oxygen can be toxic at higher than physiological concentrations, the mechanism by which oxygen regulates colony formation of cells *in vitro* is not well understood. We and others have hypothesized that oxygen toxicity may be important in regulation of colony growth of tumour cells. However, direct experimental evidence has been lacking. The data presented in Table II for the first time (to our knowledge) provide experimental evidence of the protective role of superoxide dismutase against toxic effects of superoxide anion at higher than physiological oxygen concentrations on EAT cell colony growth. Non-specific effects of superoxide dismutase were excluded by the observation that heat inactivated superoxide dismutase had no effect on PE of EAT cells.

Superoxide anion is reported to cross cell membranes (Lynch & Fridovich, 1978) and hence a gradient of superoxide anion may be formed between the intracellular and extracellular compartments. This gradient would result in intracellularly generated superoxide anion being shifted extracellularly. Therefore, the protective effect observed with superoxide dismutase may occur either intracellularly or with the dismutation of superoxide anion extracellularly. Protection extracellularly may also occur at the level of the cell membrane. The observation that a 2 h incubation with superoxide dismutase and washing out excess enzyme was effective as was a continuous exposure in overcoming the toxic effects of 20% $O_2$ suggests that superoxide dismutase may be capable of functioning extracellularly at the cell membrane or that some superoxide dismutase enters the cell as suggested by Petkau *et al.* (1982).

Differences in endogenous superoxide dismutase levels and type between murine and human tumours (Sahu *et al.*, 1977; Oberley & Buettner, 1979; Marklund *et al.*, 1983) may help to explain the quantitative differences observed with the effect of oxygen level in regulating PE of tumour cells. Endogenous superoxide dismutase levels may be important in overcoming oxygen toxicity as suggested by the observation that DDC, an inhibitor of superoxide dismutase, decreased EAT cell survival at 0.1 and 10% $O_2$ in a dose and time dependent manner. This effect of DDS was observed at low concentrations. In a preliminary experiment we were able to demonstrate that DDC does inhibit the superoxide dismutase activity of EAT cells treated for 1 h. However, it is not clear from the data at 0.1 and 10% $O_2$ whether this effect of DDC is a specific or non-specific effect, since differences in cell survival were not seen.

The antioxidants, 2-ME and GSH are known to react directly with superoxide anion (Asada & Kanematsu, 1976). Data presented in Table III suggests that these compounds also afford protection against toxic effects of 20% $O_2$ on EAT cell colony growth. In the biological system under study, it is difficult to exclude non-specific effects of these antioxidants. The observations are, however, consistent with the biological function of these agents. Although it is generally thought that GSH does not cross the cell membrane (Meister, 1983), some recent data concerning the effect of GSH on radiation sensitivity raises the possibility that GSH may enter cells (Hodgkiss & Middleton, 1983). Therefore, the effects of GSH on PE of EAT cells may occur either intracellularly or extracellularly. The formation of hydrogen peroxide, hydroxyl radicals and lipid peroxidation did not appear to be of major importance since chemical scavengers of these reaction products did not influence EAT cell colony formation. These data with EAT cells are similar to the results reported for the beneficial effect of GSH at 20% $O_2$ on erythroid colony forming units (Rich & Kuanek, 1982) and α-thioglycerol on granulocytic precursors (Bradley *et al.*, 1978). The results with EAT cells are different from that reported for beneficial effect of Vitamin E on erythroid cells (Rich & Kuanek, 1982).

It is possible that the end reactions of superoxide anion may be modulated by endogenous cellular levels of reducing substances, Vitamin E, peroxidase, catalase, and superoxide dismutase and may vary with cell type. The smaller increases in PE seen with human tumour cells compared to murine tumour cells may also be a function of the culture conditions since the Hamburger and Salmon assay has a number of anti-oxidants such as pyruvate, Vitamin C, and 2-ME.

In conclusion, these studies indicate that it is possible to grow clonogenic cells *in vitro* under conditions of physiologically low oxygen concentration (0.1–10%). Oxygen concentrations less than 20% enhance tumour cell plating efficiency and colony size. The optimal oxygen concentration for plating efficiency varies with the tumour studied and may be as low as 0.1%. Oxygen regulates plating efficiency of tumour cells through oxygen toxicity mediated by superoxide anion.

Supported in part by American Cancer Society Grant No. IN 112E, PHS Grant Number CA 32938, National Cancer Institute, DHHS, and BRSG S07-RR05427 and S07-RR07205, Biomedical Research Support Grant Program, Division of Research Resources, NIH. We are grateful to Dr F.H. Gardner for critical reviews of the manuscript and Dr Y. Awasthi for valuable discussions.

### References

ASADA, K. & KANEMATSU, S. (1976). Reactivity of thiols with superoxide radicals. *Agr. Biol. Chem.*, **40**, 1891.

BOAG, J.W. (1969). Oxygen diffusion and oxygen depletion problems in radiobiology. *Curr. Top. Radiat. Res.*, **5**, 141.

BOAG, J.W. (1970). Cell respiration as a function of oxygen tension. *Int. J. Radiat. Biol.*, **18**, 475.

BRADLEY, T.R., HODGSON, G.S. & ROSENDAAL, M. (1978). The effect of oxygen tension on haemopoietic and fibroblast cell proliferation *in vitro*. *J. Cell. Physiol.*, **97**, 517.

CATER, D.B. & SILVER, I.A. (1960). Quantitative measurements of oxygen tension in normal tissues and in the tumours of patients before and after radiotherapy. *Acta Rad.*, **53**, 233.

CHAPMAN, J.D., STURROCK, J., BOAG, J.W. & CROOKALL, J.O. (1970). Factors affecting the oxygen tension around cells growing in plastic Petri dishes. *Int. J. Radiat. Biol.*, **17**, 305.

COURTENAY, V.D. (1976). A soft agar colony assay for Lewis Lung tumour and B-16 melanoma taken directly from the mouse. *Br. J. Cancer*, **34**, 39.

COURTENAY, V.D. (1984). Primary cultures in tumours. In: *Mammalian Colony Regeneration Techniques* (Eds. Potten & Hendry) Edinburgh: Churchill Livingstone (In Press).

FROESE, G. (1962). The respiration of ascites tumour cells at low oxygen concentrations. *Biochem. Biophys. Acta*, **57**, 509.

GANFIELD, R.A., NAIR, P. & WHALEN, W.J. (1970). Mass transfer, storage, and utilization of $O_2$ in cat cerebral cortex. *Am. J. Physiol.*, **219**, 814.

GUPTA, V. & KRISHAN, A. (1982). Effect of oxygen concentration on the growth and drug sensitivity of human melanoma cells in soft-agar clonogenic assay. *Cancer Res.*, **42**, 1005.

HALLIWELL, B. (1978). Biochemical mechanisms accounting for the toxic action of oxygen on living organisms: The key role of superoxide dismutase. *Cell. Biol. Int. Rep.*, **2**, 113.

HAMBURGER, A.W. & SALMON, S.E. (1977). Primary bioassay of human tumor stem cells. *Science*, **197**, 461.

HODGKISS, R.J. & MIDDLETON, R.W. (1983). Enhancement of misonidazole radiosensitization by an inhibitor of glutathione biosynthesis. *Int. J. Radiat. Biol.*, **43**, 179.

IZAGUIRRE, C.A., CURTIS, J., MESSNER, H., & McCULLOCH, E.A. (1981). A colony assay for blast progenitors in non-B non-T (common) acute lymphoblastic leukemia. *Blood*, **57**, 823.

JAMIESON, D. & VAN DEN BRENK, H.A.S. (1964). Effect of electrode dimensions on tissue $pO_2$ measurements *in vivo*. *Nature*, **201**, 1227.

JAMIESON, D., & VAN DEN BRENK, H.A.S. (1965). Oxygen tension in human malignant disease under hyperbaric conditions. *Br. J. Cancer* **19**, 139.

KOLSTAD, P. (1968). Intercapillary distance, oxygen tension, and local recurrence in cervix cancer. *Scan. J. Clin. Lab. Invest.* (Suppl). **106**, 145.

LYNCH, R. & FRIDOVICH, I. (1978). Effects of superoxide on the erythrocyte membrane. *J. Biol. Chem.*, **253**, 1838.

MARKLUND, S.L., WESTMAN, N.G., LUNDGREN, E. & ROOS, G. (1982). Copper- and zinc containing superoxide dismutase, manganese containing superoxide dismutases, catalase, and glutathione peroxidase in normal and neoplastic human cell lines and normal human tissues. *Cancer Res.*, **42**, 1955.

McLIMANS, W.F., BLUMENSON, L.E. & TUNNAH, K.V. (1968). Kinetics of gas diffusion in mammalian cell culture systems. II Theory. *Biotech. Bioeng.*, **10**, 741.

MEISTER, A. (1983). Selective modification of glutathione metabolism. *Science*, **220**, 472.

MUELLER-KLIESSER, W.F. & SUTHERLAND, R.M. (1982). Oxygen tension in multicell spheroids of two cell lines. *Br. J. Cancer*, **45**, 256.

OBERLEY, L.W. & BUETTNER, G.R. (1979). Role of superoxide dismutase in cancer, a review. *Cancer Res.*, **39**, 1141.

OSGOOD, E.E. & KRIPPAEHNE, M.L. (1955). The gradient tissue culture method. *Exp. Cell Res.*, **9**, 116.

PETKAU, A., CHELACK, W.S., KELLY, K. & FRIESEN, H.G. (1982). Superoxide dismutase and radiosensitivity: Implications for human mammary carcinomas. In *Pathology of Oxygen* (Ed. Autor, A.P.) Academic Press, New York: p. 223.

RICH, I.N. & KUANEK, B. (1982). The effect of reduced oxygen tension on colony formation of erytheopoietic cells *in vitro*. *Br. J. Haem.*, **52**, 579.

RICHTER, A., SANFORD, K.K. & EVANS, V.J. (1972). Influence of oxygen and culture media on plating efficiency of some mammalian tissue cells. *J. Natl Cancer Inst.*, **49**, 1705.

SAHU, S.K., OBERLEY, L.W., STEVENS, R.H. & RILEY, E.E. (1977). Superoxide dismutase activity of Ehrlich ascites tumor cells. *J. Natl Cancer Inst.*, **58**, 1125.

SMITH, S.D., WOOD, G.W., FRIED, P. & LOWMAN, J.T. (1981). *In vitro* growth of lymphoma colonies from children with non-Hodgkins lymphoma. *Cancer*, **48**, 2612.

VAUPEL, P.W., FRINAK, S. & BICHER, H.I. (1981). Heterogeneous oxygen partial pressure of pH disruption in C3H mouse mammary adenocarcinoma. *Cancer Res.*, **41**, 2008.

WHALEN, W.J. & NAIR, P. (1967). Intracellular $pO_2$ and its regulation in resting skeletal muscle of the guinea pig. *Circ. Res.*, **21**, 251.

WHILLANS, D.W. & RAUTH, A.M. (1980). An experimental and analytical study of oxygen depletion in stirred cell suspensions. *Radiat. Res.*, **84**, 97.

WILSON, F.D., ERECINSKA, M., DROWN, C. & SILVER, I.A. (1979). The oxygen dependence of cellular energy metabolism. *Arch. Bioch. Bioph.*, **195**, 485.